# Correction: Al-Ahmad et al. Biodentine Inhibits the Initial Microbial Adhesion of Oral Microbiota In Vivo. *Antibiotics* 2023, *12*, 4

**DOI:** 10.3390/antibiotics13111069

**Published:** 2024-11-11

**Authors:** Ali Al-Ahmad, Michael Haendel, Markus Joerg Altenburger, Lamprini Karygianni, Elmar Hellwig, Karl Thomas Wrbas, Kirstin Vach, Christian Tennert

**Affiliations:** 1Medical Center, Department of Operative Dentistry and Periodontology, Faculty of Medicine, University of Freiburg, Hugstetter Str. 55, 79106 Freiburg, Germany; 2Clinic of Conservative and Preventive Dentistry, University of Zurich, Plattenstrasse 11, 8032 Zurich, Switzerland; 3Institute of Medical Biometry and Statistics, Faculty of Medicine, University of Freiburg, Stefan-Meier-Str. 26, 79104 Freiburg, Germany; 4Department of Restorative, Preventive and Pediatric Dentistry, University of Bern, Freiburgstrasse 7, 3010 Bern, Switzerland

We would like to update the readership about the procedure that led to the correction of [1]. Following publication, concerns were raised regarding the peer-review process related to the publication of this article [1]. Adhering to our standard procedure, the Editorial Office and Editorial Board investigated and determined that the contribution of one of the two reviewers did not comply with MDPI’s Guideline for Reviewers (https://www.mdpi.com/reviewers#_bookmark11), nor the expectations of the Editorial Board relating to the quality of the peer-review report. As a result, the Editorial Board decided to conduct a post-publication peer-review process, which included the recruitment of a new reviewer and re-evaluation by the Editor-in-Chief, to ensure that this publication fully complies with MDPI’s editorial process policy (https://www.mdpi.com/editorial_process). The journal was unable to reach originally listed Academic Editors (Marina Angélica Marciano, Débora C. Coraça-Huber and Bruno Martini Guimarães), for the overview of the post-publication peer-review process and final decision, and therefore the name of the Editor-in-Chief (Nicholas Dixon) was added.

This process has now been completed and has resulted in a number of updates to the original publication (listed below), the removal of the linked Journal Notice, the removal of the original “Reviewer 1” report, and the addition of a new report provided by the new reviewer on the open peer-review page connected to the article (https://www.mdpi.com/2079-6382/12/1/4/review_report).

## Text Correction

The authors wish to make the following corrections to the original publication [1]:

The authors were asked under Section 4.1.1. Inclusion criteria to provide more specific details, for example, for caries free, to write teeth caries free or individuals with caries free, and for age ≥ 18 years, individuals or subjects aged ≥ 18 years. They were asked the same for the exclusion criteria.

## Corrected Paragraph Under Section 4.1.1

4.1.1. Inclusion Criteria
-individuals aged ≥ 18 years-caries-free individuals

4.1.2. Exclusion Criteria
-individuals with known allergy to the materials or their components and/or suffering from infectious or life-threatening diseases-breastfeeding or pregnant individuals-individuals with temporary use of antibiotics in the last six months or anti-inflammatory medication within the last 30 days-individuals with serious general illnesses, such as diabetes, HIV, hepatitis B and C, acute tumor diseases, or epilepsy

The authors were asked to discuss the biofilm formation in the discussion section, since the microorganisms in oral cavity are in this form.

## Added Paragraph in the Discussion

Initial biofilm formation is affected by multiple factors, e.g., surface charge, surface energy, roughness and topography [29]. Most bacteria have been found to be negatively charged. Thus, in general, bacteria are prone to a positively charged surface, while a negatively charged surface is more resistant to bacterial adhesion. Surfaces containing certain cationic groups have antimicrobial activities and thus can kill the attached microbial cells [30]. The setting of silicate cements results in a negatively charged surface charge through an excess of calcium hydroxide formed by OH^−^ leading to a rise in the calcium concentration in the surrounding environment [31]. Roughness and topography seem to be the most important factors regarding microbial adhesion. Thus, smoothening the surface, as we performed in the present study, can reduce biofilm formation, and a roughness of R_a_ of 0.2 µm seems to be the threshold for the maximum reduction in bacterial adhesion on surfaces [32]. However, the exact effects of surface roughness on bacterial adhesion and biofilm formation vary with the size and shape of bacterial cells and other environmental factors.

The authors were asked to add the specific clinical implication of this study.

## Added Paragraph in the Conclusions

Biodentine might be the preferred material used in deep tooth cavities or the pulp chamber when they are contaminated with microorganisms.

## References

The newly added references are listed below:
29.Song, F.; Koo, H.; Ren, D. Effects of Material Properties on Bacterial Adhesion and Biofilm Formation. *J. Dent. Res.* **2015**, *8*, 1027–1034. https://doi.org/10.1177/0022034515587690.30.Campoccia, D.; Montanaro, L.; Arciola, C.R. A review of the biomaterials technologies for infection-resistant surfaces. *Biomaterials* **2013**, *34*, 8533–8554. https://doi.org/10.1016/j.biomaterials.2013.07.089.31.Solanki, N.P.; Venkappa, K.K.; Shah, N.C. Biocompatibility and sealing ability of mineral trioxide aggregate and biodentine as root-end filling material: A systematic review. *J. Conserv. Dent.* **2018**, *1*, 10–15. https://doi.org/10.4103/JCD.JCD_45_17.32.Ionescu, A.; Wutscher, E.; Brambilla, E.; Schneider-Feyrer, S.; Giessibl, F.J.; Hahnel, S. Influence of surface properties of resin-based composites on in vitro *Streptococcus mutans* biofilm development. *Eur. J. Oral Sci.* **2012**, *5*, 458–645. https://doi.org/10.1111/j.1600-0722.2012.00983.x.

With this correction, the order of some references has been adjusted accordingly. The authors state that the scientific conclusions are unaffected. This correction was approved by the Academic Editor. The original publication has also been updated.
